# Nitrate Uptake and Use Efficiency: Pros and Cons of Chloride Interference in the Vegetable Crops

**DOI:** 10.3389/fpls.2022.899522

**Published:** 2022-06-16

**Authors:** Petronia Carillo, Youssef Rouphael

**Affiliations:** ^1^Department of Environmental, Biological and Pharmaceutical Sciences and Technologies, University of Campania Luigi Vanvitelli, Caserta, Italy; ^2^Department of Agricultural Sciences, University of Naples Federico II, Naples, Italy

**Keywords:** N fertilization, nitrate sensing, chloride toxicity, chloride beneficial macronutrient, salinity eustress

## Abstract

Over the past five decades, nitrogen (N) fertilization has been an essential tool for boosting crop productivity in agricultural systems. To avoid N pollution while preserving the crop yields and profit margins for farmers, the scientific community is searching for eco-sustainable strategies aimed at increasing plants’ nitrogen use efficiency (NUE). The present article provides a refined definition of the NUE based on the two important physiological factors (N-uptake and N-utilization efficiency). The diverse molecular and physiological mechanisms underlying the processes of N assimilation, translocation, transport, accumulation, and reallocation are revisited and critically discussed. The review concludes by examining the N uptake and NUE in tandem with chloride stress and eustress, the latter being a new approach toward enhancing productivity and functional quality of the horticultural crops, particularly facilitated by soilless cultivation.

## Introduction

For agricultural cropping systems, nitrogen (N) fertilization has been represented as a useful tool to improve plant growth, yield components, and quality. The high-energy cost for N fertilizer synthesis as well as its intrinsic mobility in the complex atmosphere–plant–soil system have highlighted the environmental drawbacks of the unsustainable N use (Keeney and Hatfield, 2001; Rothstein, 2007; Garnett et al., 2009; Chen K. E. et al., 2020; Bijay and Craswell, 2021). In this respect, there is a growing interest in the improvement of nitrogen use efficiency (NUE), especially for the horticulture crops, which are notoriously characterized by excessive and unjustified N “consumption” (Carillo et al., 2019a). NUE depends on the N-uptake efficiency, the amount of N consumed by the crop per unit of available N, the N utilization efficiency, and the harvestable product per unit of N uptake (Moll et al., 1982; Sisson et al., 1991). Nitrate (NO_3_^–^) is the main source of N in plants, but its concentration in the soil can fluctuate dramatically due to either time or space, thus becoming one of the main factors limiting the crop growth and development (Gojon et al., 2011). In fact, NO_3_^–^ concentration is highly variable, ranging from 0.1 to 10 mM, depending on the soil process dominating at that time point, such as bursts of nitrification, leaching process intensification, or fertilization (Crawford and Glass, 1998; Miller et al., 2007). The importance of NO_3_^–^ is attributed to its signaling role, which can trigger specific responses at different levels (cellular, biochemical, and molecular) and induce gene expression regulating its own uptake. NO_3_^–^ assimilation, driven by the enzyme nitrate reductase (NR), involves the uptake of NO_3_^–^, its reduction to nitrite (NO_2_^–^) and then to ammonium (NH_4_^+^), and finally its incorporation into the organic compounds. Plants can adjust their capacity to acquire NO_3_^–^ by reshaping the root architecture to enhance NO_3_^–^ uptake and by modulating the NO_3_^–^-assimilation pathway. The comprehension of this nutrient acquisition response mechanism could help to improve the plants’ NUE. Significantly, if NO_3_^–^ uptake exceeds the assimilative capacity of the plant, it can accumulate in the plant tissues, which in the case of leafy vegetables can be unsafe (Gupta et al., 2017). Human gastrointestinal metabolism reduces NO_3_^–^ to NO_2_^–^, which, when reacting with N-based organic compounds, can form compounds with recognized carcinogenic action (Santamaria, 2006; Colla et al., 2018). As a preventive measure, the European Commission regulations n° 1881/2006 and 1258/2011 have *de facto* set a maximum NO_3_^–^ threshold for leafy vegetables, such as spinach (*Spinacia oleracea* L.), lettuce (*Lactuca sativa* L.), and rocket (*Eruca vesicaria* L.). In this perspective, more and more studies have focused not only on the role of genetics, physiological status, timing, and the form of N application but also especially on the search for alternative horticultural and agronomic practices that can limit NO_3_^–^ accumulation without compromising the product performance, such as the use of salinity as eustressor (Rouphael et al., 2018), the modified intermittent nutrient film technique (NFT) (Tabaglio et al., 2020), or the hydroponics to constantly monitor the nutrient solution (Ciriello et al., 2021). Therefore, it is not surprising that soilless systems, due to the potential to modulate and control a plant’s nutritional status, could be used to induce positive stress (eustress) that can limit the excessive accumulation of NO_3_^–^ (Lucini et al., 2016; Rouphael and Kyriacou, 2018). NO_3_^–^ uptake can be affected by Cl^–^ that can indirectly interfere with the NO_3_^–^-uptake mechanisms by decreasing the internal demand for NO_3_^–^ and consequently its utilization efficiency. However, although NO_3_^–^-uptake pathways and Cl^–^-inhibitory effects are well-documented, the possible implications of their interaction and the resulting impacts (negative or positive) on vegetables have not been clarified. Maximizing NUE in future agricultural systems requires an understanding of the diverse genetic and physiological mechanisms underlying the processes of N assimilation, translocation, transport, accumulation, and reallocation. In fact, a complete understanding of these processes will allow the implementation of efficient strategies. Our review aimed to elucidate these crucial mechanisms that are directly involved in N metabolism and also describe the possibility of using chloride eustress to improve NUE while reducing NO_3_^–^ accumulation in the leafy vegetables.

## Nitrogen Use Efficiency

The basic concept of NUE describes the yield of a harvestable product (dry matter) per unit N available or even the grain yield (kg) per unit (kg) of total available N (applied N + soil mineral N) (Hirose and Kitajima, 1986). However, NUE depends not only on the plant N uptake efficiency (NUpE, kg kg^–1^) but also on its assimilation and translocation and, in aged plants, on recycling and remobilization and therefore on N utilization efficiency (NUtE, kg kg^–1^) (Moll et al., 1982; Masclaux-Daubresse et al., 2010; Xu et al., 2012; Hawkesford et al., 2014). NutE, in particular, concerns the capacity of a certain species or cultivar to convert the assimilated/remobilized N in biomass (grain, leaves, and fruit) and, in the end, will be equal to the harvestable product per unit of N consumed by a crop (Todeschini et al., 2016). Indeed, an efficient N application helps decrease N losses from the soil–plant system, increasing NUpE and NutE, and therefore the amount of agricultural output (Li et al., 2018).

In the last decades, the increase of NUE (NUpE + NUtE) has been considered a focus to reduce the use of N fertilizers and minimize their cost and environmental impact (Hirose, 2011). In fact, plants can absorb only 30–50% of the approximately 110 million tons of N fertilizers spread over the fields every year, losing the rest due to surface runoff, leaching, volatilization, microbial consumption, and denitrification (Garnett et al., 2009; Chen K. E. et al., 2020).

In this scenario, the horticulture production of vegetable crops, which have high economic and nutritious value, entails the highest use of chemicals (in particular N) per unit area than any other agricultural system, causing high costs and environmental pollution (Carillo et al., 2019a). Moreover, the horticultural production systems are more prone to N losses than grain crop systems because of the higher rates of N fertilizer used and the shallow root systems of the horticultural plants compared to arable plants (Cameron et al., 2013). Therefore, there is a high interest in the field of horticultural science in reducing N inputs and improving NUE for the production of vegetable crops by selecting new genotypes, mostly by exploiting genetic variation in the available germplasm, understanding the physiological mechanisms involved in these mechanisms, and finding new management practices for the existing crops. The increase of NUE by only 1% may save USD$1.1 billion (Van Oosten et al., 2019) and can also reduce nitrous oxide emissions.

## Nitrate Effect on the Root Development

Greenhouse horticulture is the best example of excessive NO_3_^–^/resource intensive agriculture, requiring the highest use of N/NO_3_^–^ per unit area compared to other agricultural systems, resulting in high financial costs and environmental risks for the high N losses (Carillo et al., 2019a). However, until now, horticultural plants have been grown nearly under non-limiting N conditions, because the attempts to reduce N fertilization resulted in reduced plant growth and poor yield (Masclaux-Daubresse et al., 2010). N, in fact, is of pivotal importance in the plant’s metabolism. NR, the first enzyme in the NO_3_^–^ assimilation pathway catalyzing the reduction of NO_3_^–^ in NH_4_^+^, is strictly dependent on the plant NO_3_^–^ flux from roots and in general on NO_3_^–^ availability at the cellular level (Carillo et al., 2005; Annunziata et al., 2017). This enzyme represents the limiting step in the overall process of plant growth and productivity (Kaiser et al., 1999). NO_3_^–^ is required for full levels of NR gene expression, as signals from N metabolism play an important role in inducing the expression of the NR gene Nia (Oaks, 1974).

Plants can modulate their NO_3_^–^ uptake, storage, and assimilation according to the internal and external spatio-temporal changes in N status by modulating the type, number, spatial pattern, and affinity of hundreds of genes expressing NO_3_^–^ transporters (Forde, 2002; Orsel et al., 2002; Bouguyon et al., 2012; Boer et al., 2020) in addition to extensively re-shaping the root system architecture (RSA) (Aibara and Miwa, 2014). In fact, low N status can upregulate NO_3_^–^ uptake system (Nacry et al., 2013) and modify plant root architecture, increasing root length, density, and branching, thus resulting in a “nutrient acquisition response” improving NUE. Depending on its availability and distribution, NO_3_^–^ can have both positive and negative effects on the development and growth of the lateral roots (Zhang et al., 1999; Nacry et al., 2013). In fact, it was demonstrated that, when *Arabidopsis* roots were exposed to a locally concentrated supply of NO_3_^–^, there was no increase in the lateral roots numbers but a 2-fold increase of elongation caused by an enhanced cell production in the lateral root meristem (Zhang et al., 1999). Other locally applied N sources, like NH_4_^+^, can promote lateral root branching but not elongation (Lima et al., 2010), proving that NO_3_ acts as a signal probably interacting and/or interfering with auxin response pathways (Zhang et al., 1999). The phenotypic plasticity of plants, which makes roots to grow preferentially toward NO_3_^–^-richer zones at low NO_3_^–^, is termed “root foraging”; whereas NO_3_^–^ has been defined as an “environmental morphogen” for its ability to modulate the root architecture and root foraging (Giehl and von Wirén, 2014; Guan et al., 2014; Boer et al., 2020). The foraging response at low NO_3_^–^ that entails root growth is exerted through the overexpression of the (i) TRYPTOPHAN AMINOTRANSFERASE-RELATED PROTEIN 2 (TAR2), involved in local auxin biosynthesis; (ii)WALL-ASSOCIATED KINASE 4 (WAK4); and iii) MULTIDRUG RESISTANCE4/P-GLYCOPROTEIN 4 (MDR4/PGP4), a downstream transporter of auxin (Giehl and von Wirén, 2014; Ma et al., 2014; Sun et al., 2017). Cytokinin signaling is also involved in the NO_3_^–^ foraging response; in fact, this hormone is synthetized in a NO_3_^–^-dependent manner and is translocated to shoot, where it induces the expression of the genes involved in a complex long-distance root–shoot–root signaling network entailing root foraging (Poitout et al., 2018; Roy, 2018).

Giehl and von Wirén (2014) observed a continuous root growth response when plants grew in a homogeneous external NO_3_^–^ concentration but a repressing surviving response at severely low NO_3_^–^ concentration. This surviving response is regulated by the N-responsive CLAVATA3/ESR-related (CLE) signaling peptides and their receptor protein CLAVATA1 (CLV1) (Araya et al., 2014). Moreover, since there is upregulation of the dual-affinity NO_3_^–^ transporter NRT1.1 in the lateral roots at a very low NO_3_^–^ concentration, which acts as an auxin importer at low external NO_3_^–^ levels, this determines a shootward movement of auxin that strongly decreases the concentration of this hormone in the lateral root tissues, consequently inhibiting the lateral root growth (Krouk et al., 2010; Giehl and von Wirén, 2014). At high NO_3_^–^ concentrations, NRT1.1 is not able to transport auxin, thus does not decreasing the lateral root growth (Krouk et al., 2010); while it again starts to transport auxin at very high levels of NO_3_^–^, stimulated by a signaling pathway modulated by the (i) protein AUXIN SIGNALING F-BOX 3 (AFB3), (ii) NAC4 transcription factor, and (iii) OBF Binding Protein 4 (OBP4), exerting a repression response, which determines inhibition of root growth, cell elongation, and differentiation (Vidal et al., 2013).

On the contrary, NRT2.1 in rice regulates a NO_3_^–^-dependent root elongation involving auxin transport to roots; this mechanism appears related to the NO_3_^–^-dependent production of NO that upregulates PIN-FORMED 1 (PIN1), a key mediator of basipetal polar auxin transport in the cell roots, which promotes a reorientation of auxin transport toward the tip of the newly developing root (Naz et al., 2019).

## Nitrate Transporters and Sensing

NO_3_^–^ is consumed by the roots and mobilized to the other organs by NO_3_^–^ transporters, which display a bi-phasic pattern (Crawford and Glass, 1998). In the low concentration range, a high-affinity transport system (HATS) can uptake NO_3_^–^ from the soil at concentrations of 10-250 μM with an activity fitting the Michaelis-Menten kinetic model (Filleur et al., 2001). The HATS has both a constitutive component (cHATS) and a NO_3_^–^-inducible component (iHATS), whose *V*_*max*_ was 30-fold higher than the cHATS one (Zhuo et al., 1999; Li et al., 2007; Gao et al., 2019). Whereas, starting from the concentration of about 0.5 mM, NO_3_^–^ uptake is performed by two high-affinity transport systems (LATS) that substitute/superimpose the HATS: one is constitutive (cLATS), which does not show saturation even at 50 mM external NO_3_^–^, and the other is inducible (iLATS) (Liu et al., 1999; Zhuo et al., 1999; Forde, 2000). Both HATS and LATS proceed thermodynamically uphill since the uptake of NO_3_^–^ is depressed or inhibited by processes that decrease or inhibit the synthesis of ATP and proteins (Peuke and Jeschke, 1998). NO_3_^–^ uptake, in fact, is an energy-dependent transport consistent with NO_3_^–^: 2 H^+^ symport that requires the creation of an H^+^ electrochemical gradient, generated by a proton translocation coupled to ATP hydrolysis (Crawford and Glass, 1998; Forde, 2000). In addition, plants show an inducible NO_3_^–^-efflux system with a much slower turnover rate than the uptake system, which requires RNA and protein synthesis (Aslam et al., 1996).

The first NO_3_^–^-transporter gene identified in plants belonging to LATS was the *AtNRT1.1* gene, originally named *CHLORINA1* (*CHL1*) because it was associated with chloride (Cl^–^) sensitivity in Arabidopsis (Huang et al., 1996; Liu et al., 1999) but now known as *AtNPF6.3*. Subsequently, it was found that NRT1.1 also functions as a HAT at low NO_3_^–^levels; therefore, it is a dual-affinity transporter that can facilitate NO_3_^–^ uptake at concentrations ranging from micromolar to millimolar (Liu et al., 1999). NRT1.1 has been demonstrated to contribute to over 75% of the high-affinity NO_3_^–^ uptake in plants (Wang et al., 1998). It is involved in the NO_3_^–^ uptake and transport (Liu et al., 1999), auxin transport activity (Krouk et al., 2010; Bouguyon et al., 2016), NO_3_^–^-modulated root development (Bouguyon et al., 2016; Albornoz et al., 2018), NO_3_^–^ sensing (Miller et al., 2007), and growth improvement under N deficiency stress (Ho et al., 2009; Bouguyon et al., 2012, 2016). NRT1.1 has been defined as a moonlighting protein because it performs more than a single function (Fichtner et al., 2021) and also as a transceptor because it has transporter and receptor roles (Gojon et al., 2011). The intermediates of the oxidative pentose phosphate pathway regulate its expression and consequently root N levels (Lejay et al., 2008; Table 1).

**TABLE 1:** Genes related to N uptake translocation and assimilation.

Genes	Functions	References
*AtNRT1.1 (AtCHL1/AtNPF6.3)*	First NO_3_^–^ transporter identified in plants belonging to NO_3_^–^ low-affinity transport system (LATS). Associated with chlorate sensitivity and therefore also named CHLORINA1 (*CHL1*). Defined as *moonlighting protein* because it performs more than a single function and as *transceptor* because it has transporter and receptor roles. Dual-affinity transporter able to facilitate NO_3_^–^ uptake at concentrations ranging from micromolar to millimolar. In *A. thaliana*, it is involved in (i) NO_3_^–^ uptake and transport, (ii) auxin transport activity, (iii) NO_3_^–^-modulated root development, (iv) NO_3_^–^ sensing, (v) growth improvement under nitrogen deficiency stress. It inhibits lateral root growth at low NO_3_^–^ by inducing basipetal auxin transport out of these roots. Oxidative pentose phosphate pathway intermediates regulate its expression and root N levels.	Tsay et al., 1993; Liu et al., 1999; Lejay et al., 2008; Ho et al., 2009; Krouk et al., 2010; Bouguyon et al., 2012, 2015, 2016; Mounier et al., 2014; Sakuraba et al., 2021
*LeNRT1.1*	Involved in NO_3_^–^ uptake in grafted *Lycopersicum esculentum* under high N demand	Albornoz et al., 2018
OsNRT1.1A/OsNPF6.3	It upregulates the expression of genes involved in N utilization (both for NO_3_^–^ and NH_4_^+^) and flowering shortening grain yield and maturation in *Oryza sativa*.	Wang and Tsay, 2011
*OsNRT1.1B*	It is involved in (i) NO_3_^–^ signal transduction from the plasma membrane to the nucleus and (ii) integration of NO_3_^–^ and phosphate signaling networks, (iii) regulation of root microbiota to improve N mineralization in soil, thus mediating the plant–microbe interactions in *Oryza sativa.*	Fan et al., 2016; Hu et al., 2019; Zhang et al., 2019
*AtNRT1.2/AtNPF4.6*	Inducible component of LATS in *A. thaliana*. Function as (i) ABA importer at the site of ABA biosynthesis, (ii) regulator of stomatal aperture in inflorescence stems, and (iii) regulator of ABA response during germination and seedling development.	Li et al., 2020; Zhang et al., 2021
*GmNRT1-2*	Putative LATS NO_3_^–^ transporter downregulated after a short exposure to NO_3_^–^ and/orNH_4_^+^ medium and upregulated during nitrate-limitation (likely a high-affinity nitrate transporter) in *Glycine max.*	Yokoyama et al., 2001
*LeNRT1.2*	Involved in NO_3_^–^ uptake in ungrafted *Lycopersicum esculentum* plants	Albornoz et al., 2018
*AtNRT1.3/AtNPF6.4*	Nitrate transporter specifically functioning in parenchymal tissues, related to polyamine transport or metabolism in *Arabidopsis*.	Tong et al., 2016
*GmNRT1.3*	Putative LATS NO_3_^–^ transporter in *G. max.*	Yokoyama et al., 2001
*MtNRT1.3*	Dual-affinity transporter involved in NO_3_^–^ and ABA transport in *Medicago truncatula*.	
*AtNRT1.4*	Regulation of leaf NO_3_^–^ homeostasis and leaf development in *A. thaliana.*	Chiu et al., 2004
*AtNRT1.5*	Involved in xylem loading of NO_3_^–^ from root to shoot transport of nitrate.	Lin et al., 2008
*AtNRT1-6*	Role in delivering NO_3_^–^ from the maternal tissue to the developing embryo of *A. thaliana.*	Almagro et al., 2008
*AtNRT1.7*	Responsible for source to sink remobilization of NO_3_^–^ *via* phloem from older to younger leaves of *A. thaliana.*	Fan et al., 2009
*AtNRT1.8*	Present in the plasma membrane of xylem parenchyma cells of *A. thaliana*, it is involved in the uptake of NO_3_^–^ from the xylem sap into the xylem parenchyma cells. Function in Cd^2+^ tolerance.	Li et al., 2010
*GmNRT1.7a, GmNRT1.7b*	Putative nitrate transporter is responsible for NO_3_^–^ translocation from leaves to seeds in *G. max.*	Inoue et al., 2014
*AtNRT1.9*	Expressed in the companion cells of the root phloem of *A. thaliana*, it loads NO_3_^–^ into the root phloem and enhances downward NO_3_^–^ transport in roots.	Wang and Tsay, 2011
*AtNRT2.1 (ACH1)*	Nitrate transporter identified in *A. thaliana* belonging to NO_3_^–^ high-affinity transport system (HATS), regulated by external NO_3_^–^, N starvation, sucrose, and light (circadian or diurnally regulation). It is downregulated by NH_4_^+^, amino acids, N-metabolites resulting from NO_3_^–^ reduction, and dark. It does not mediate transport on its own but functions as a dual component transporter with NTR3.1. It inhibits lateral root initiation under high sucrose/low NO_3_^–^ conditions. It works as a central player in the integration of C- and N-metabolism and is transcriptionally and post-transcriptionally regulated by C- and N-derived metabolites. Oxidative pentose phosphate pathway intermediates regulate its expression and consequently root N levels. NRT2.1, NRT2.2, and NRT2.4 are required to ensure optimal adaptation to N limitation.	Lejay et al., 1999, 2008; Filleur et al., 2001; Girin et al., 2007; Kiba et al., 2012; de Jong et al., 2013; Fichtner et al., 2021
*OsNRT2.1*	Involved in NO_3_^–^-dependent root elongation in *O. sativa* by regulating polar auxin transport to roots.	Naz et al., 2019
*NRT2.2 (ACH2)*	It only functions as a dual component transporter with NAR2.1 importing NO_3_^–^ with high affinity. Plants over-expressing NRT2.2 increase their growth under low NO_3_^–^ conditions.	Filleur et al., 2001
*OsNRT2.3*	Functioning as a dual component transporter with NAR2.1, it undergoes circadian regulation with a peak in the middle of the morning and at the end of the light period and downregulation by NH_4_^+^ and NH_4_^+^-derived metabolites. It has a key role in long-distance NO_3_^–^ transport from roots to shoots, particularly at low external NO_3_^–^ supply. Its co-overexpression with *OsNAR2.1* may increase rice yield and nitrogen use efficiency. *OsNRT2.3a* plays a key role in root to shoot NO_3_^–^ translocation under N limiting conditions.	Feng et al., 2011; Yan et al., 2011; Tang et al., 2012; Chen J. et al., 2020
*LeNRT2.3*	Double role in NO_3_^–^ uptake and long-distance transport in tomato. Present in the plasma membranes and involved in NO_3_^–^ uptake in root and transport from roots to shoots. Its overexpression determines high biomass and fruit weight.	Fu et al., 2015
*AtNRT2.4*	Role in both roots and shoots under N starvation, transferring NO_3_^–^ from stored pools to cytoplasm.	Kiba et al., 2012
*AtNRT2.5*	Nitrate transporter involved in (i) the phloem loading of NO_3_^–^ to shoots in mature plants under long-term N starvation conditions, (ii) transfer of NO_3_^–^ from stored pools to the cytoplasm, (iii) induction of NO_3_^–^ inducible genes in roots previously deprived of NO_3_^–^. Role in the NO_3_^–^ uptake-independent plant growth promotion and lateral root response to the rhizospheric *Phyllobacterium*.	Kechid et al., 2013; Lezhneva et al., 2014; Kotur and Glass, 2015
*AtNRT2.6*	Strongly upregulated upon inoculation with the plant growth-promoting rhizobacteria *Phyllobacterium.*	Kechid et al., 2013
*AtNRT2.7*	Localized to the vacuole membrane has a key role in NO_3_^–^ accumulation in the seeds. Downregulated by imbibition.	Chopin et al., 2007
*NPF5.11, NPF5.12 and NPF5.16*	Vacuole nitrate efflux transporters are tonoplast-localized, expressed preferentially in root pericycle cells and xylem parenchyma cells.	He et al., 2017
*AtNIA1*	NADH-Nitrate reductase 1 is a key enzyme that accounts for 10–15% of NO_3_^–^ reductive assimilation in shoots. When mutated, it confers resistance to the herbicide chlorate. It is activated by NO_3_^–^ and sumoylation. It is involved in the nitric oxide biosynthetic process. NIA1 transcript is present throughout the life cycle of *A. thaliana* being predominantly active in leaves.	Wilkinson and Crawford, 1993; Vitor et al., 2013; Olas and Wahl, 2019; Wang et al., 2021
*AtNIA2*	NADH-Nitrate reductase 2 is responsible for 90% of the total nitrate reductase activity in seedlings. NIA2 complements NIA1 in the same organs and tissues. It is involved in (i) NO_3_^–^ assimilation, (ii) nitric oxide biosynthesis, (iii) response to light (by phytochrome and blue light photoreceptors), and (iv) response to symbiotic fungi. Sumoylation increases enzyme activity and promotes NO_3_^–^ assimilation. Its transcript is present throughout the life cycle of *A. thaliana* being predominantly active in meristematic tissues.	Wilkinson and Crawford, 1991; Sherameti et al., 2005; Olas and Wahl, 2019; Wang et al., 2021
*AtNir1*	Nitrite reductase catalyzes the six-electron reduction of NO_2_^–^ to NH_4_^+^. NiR protein pool is almost exclusively constituted by NiR1, whose expression is induced by nitrate but unaffected by light. A key target in regulating nitrogen assimilation and NO homeostasis by being relevant to the control of both plant growth and performance under stress conditions. Since most higher crop plants have only this isoform of NiR, the modulation of its function may represent a relevant agrobiotechnological target.	North et al., 2009; Konishi and Yanagisawa, 2010; Costa-Broseta et al., 2020
*LeNiR2*	Predominant NiR isoform in tomato seedlings cotyledons. Response to light mediated by phytochrome and blue-light photoreceptors.	Becker et al., 1992; Migge et al., 1998

AtNRT2.1 (ACH1) is another HAT regulated by external NO_3_^–^ (Filleur et al., 2001), N starvation (Li et al., 2007), sucrose, and light (circadian or diurnal regulation) (Lejay et al., 1999; de Jong et al., 2013). It is downregulated by NH_4_^+^, amino acids, N-metabolites resulting from NO_3_^–^ reduction, and dark (de Jong et al., 2013). It does not mediate transport on its own but functions as a dual-component transporter with NTR3.1 (Tong et al., 2005). It inhibits the lateral root initiation under high-sucrose/low-NO_3_^–^ conditions (Little et al., 2005). It works as a central player in the integration of C- and N-metabolisms and is transcriptionally and post-transcriptionally regulated by C- and N-derived metabolites (de Jong et al., 2013). NRT2.1, NRT2.2, and NRT2.4 are required to ensure an optimal adaptation to N limitation (Kiba et al., 2012). OsNRT2.1 is involved in NO_3_^–^-dependent root elongation in *Oryza sativa* by regulating polar auxin transport to the roots (Naz et al., 2019; Table 1).

NRT2.2 (ACH2) acts as a dual-component transporter with NAR2.1 importing NO_3_^–^ with high affinity; plants over-expressing NRT2.2 increase their growth under low NO_3_^–^ conditions (Filleur et al., 2001). NRT2.3 acts as a dual-component transporter with NAR2.1 undergoing circadian regulation with a peak in the middle of the morning and at the end of the light period and downregulation by NH_4_^+^ and NH_4_^+^-derived metabolites (Feng et al., 2011). It has a key role in long-distance NO_3_^–^ transport from roots to shoots, particularly at low external NO_3_^–^ supply (Fu et al., 2015). Its co-overexpression with NAR2.1 may increase rice yield and NUE (Chen J. et al., 2020). OsNRT2.3a plays a key role in root to shoot NO_3_^–^ translocation under N-limiting conditions (Tang et al., 2012). The overexpression of OsNRT2.3b has also been correlated with high grain yield and NUE in rice (Sandhu et al., 2021; Table 1).

NO_3_^–^ is an important signal molecule that can trigger a range of responses at the molecular, biochemical, and cellular levels in the plant roots (Bouguyon et al., 2012). NO_3_^–^ induces the expression and/or the transcription of the genes involved in its own uptake (e.g., *HATS*), whereas the addition of NH_4_^+^ or glutamine leads to a decrease in transcripts for the transporter system (Sanz-Luque et al., 2015). NO_3_^–^ is also an important determinant for the induction of the NR genes *NIA*, and for the stability of the NR transcripts (Galangau et al., 1988; Foyer et al., 1998; Konishi and Yanagisawa, 2013).

In particular, the *NIA1* encodes the cytosolic NADH-NR1, an enzyme present throughout the life cycle of plants being predominantly active in leaves in which it accounts for 10–15% of NO_3_^–^ reductive assimilation (Olas and Wahl, 2019). When the *NIA1* is mutated, it confers resistance to the herbicide chlorate (Wilkinson and Crawford, 1993). The biosynthesis of NADH-NR1 is activated by NO_3_^–^ sumoylation (modulation by a small ubiquitin-related modifier, SUMO) and cytokinins (Yu et al., 1998; North et al., 2009; Park et al., 2011). *NIA2* encodes for an NADH-NR 2 and is responsible for 90% of the total NR activity in seedlings. NIA2 complements NIA1 in the same organs and tissues and is involved in NO_3_^–^ assimilation (Wilkinson and Crawford, 1991; Olas and Wahl, 2019), in response to light mediated by phytochrome and blue-light photoreceptors (Migge et al., 1998; Lillo and Appenroth, 2001), and in response to symbiotic fungi (Sherameti et al., 2005). Its transcript is present throughout the life cycle of plants being predominantly active in the meristematic tissues (Olas and Wahl, 2019). Both NIA1 and NIA2 are critical in nitric oxide (NO) production and are involved in the abscisic acid (ABA)-induced stomatal closure (Sun et al., 2015; Zhao et al., 2016; Costa-Broseta et al., 2020; Table 1). In rice, the NO produced by the NR pathway has a key role in improving the NUE by increasing the lateral roots initiation and inorganic N uptake rate, allowing plants to adjust plant NO_3_^–^ acquisition to the fluctuating availability (Sun et al., 2015). The NR-dependent NO production is also critical for disease resistance; in fact, NO, in combination with H_2_O_2_, has a very efficient and cost-effective microbicidal effect that can reduce the energy expenditure associated with salicylic acid (SA)-mediated defense response (Vitor et al., 2013).

The supply of NO_3_^–^ and/or metabolites formed during the NO_3_^–^ assimilation can activate phosphoenolpyruvate carboxylase (PEPCase) and inactivate the sucrose phosphate synthase (SPS) activity (Scheible et al., 1997). Nitrate can also induce the expression of genes and enzymes involved in the NH_4_^+^ assimilation (e.g., root glutamine synthetase, GS) and increase the synthesis of organic acids which are useful as carbon skeletons for amino acids synthesis or as counter-anions (Scheible et al., 1997; Garnica et al., 2010; Sanz-Luque et al., 2015). Glutamine and NH_4_^+^ have roles in the feedback repression of NO_3_^–^ uptake and assimilation (Stitt et al., 2002; Masclaux-Daubresse et al., 2010; Nacry et al., 2013). However, the presence of NR and/or its metabolism’s products are not required for NO_3_^–^ sensing (Scheible et al., 1997). Fluctuations in the levels of NO_3_^–^ may affect the biosynthesis of carbohydrates and *vice versa*; in fact, NO_3_^–^ may inhibit the synthesis of starch (Foyer and Paul, 2001; Stitt et al., 2002; Fichtner et al., 2021) and modulate the carbohydrates allocation and development system (Wang et al., 2012; O’Brien et al., 2016).

Light may stimulate the root uptake of NO_3_^–^ by a modulation effect exerted by recent photosynthates transported from shoots to roots, with a diurnal rhythm of NO_3_^–^ peaking during the light period, while getting a minimum in the dark (Peuke and Jeschke, 1998; Lejay et al., 1999; Ruffel et al., 2014). Sucrose may replace the light-mediated response on NO_3_^–^ uptake (Zhou et al., 2009). The extent of NO_3_^–^ uptake and the modulation of the pH of the xylem sap may have a role in stomatal regulation by the delivery of ABA to guard cells (Gloser et al., 2020).

## Nitrate Transport, Accumulation, and Re-Allocation

Nitrate can be accumulated or reduced and assimilated into amino acids in roots and/or in shoots, after being transported *via* xylem. If NO_3_^–^ remains in the cytoplasm, it is rapidly reduced to NO_3_^–^ and then assimilated; thus, the concentration of NO_3_^–^ in plant tissues is modulated by the ratio of the distribution of NO_3_^–^ between the cytoplasm and the vacuole (Liang and Zhang, 2020). *Arabidopsis thaliana* tonoplast Cl^–^ channel an (AtCLCa) accumulation of NO_3_^–^, specifically in the vacuole and behaves as a NO_3_^–^/H^+^ exchanger, actively mediating the relative amounts of cytoplasm and vacuole NO_3_^–^ reservoirs (De Angeli et al., 2006). Han et al. (2016) demonstrated that a decrease in the vacuolar sequestration capacity of NO_3_^–^ in the roots of *Brassica napus* may enhance the transport to shoots contributing to the increase in NUE by promoting NO_3_^–^ allocation to the aerial parts. Nitrate stored in the vacuole can be used for assimilation, serving as a reservoir to support the growth when the external N supply gets limited (Leij et al., 1998).

Nitrate remobilization from vacuoles to other plant tissues/organs is a key component of NUE (Chen K. E. et al., 2020). NPF5.11, NPF5.12, and NPF5.16 vacuolar NO_3_^–^ efflux transporters in *Arabidopsis* may act for up taking NO_3_^–^ from the vacuoles to the cytosol, thus functioning as important modulators of NO_3_^–^ allocation between roots and shoots (He et al., 2017). Thus, the finding that the cytosolic concentration of NO_3_^–^ is maintained constant and that surplus NO_3_^–^ is accumulated in the vacuole implies that NO_3_^–^ regulates the activity of the transport system on the tonoplast (Scheible et al., 1997). Moreover, since xylem transport is controlled by transpiration, expanded leaves that have a larger transpiration surface may obtain higher amounts of NO_3_^–^ (Chen K. E. et al., 2020). The low-affinity NO_3_^–^ transporters in *Arabidopsis*, NRT1.11 and NRT1.12 (also known as NPF1.2 and NPF1.1, respectively) expressed in the companion cells of the source leaves, are responsible for NO_3_^–^ transport from the xylem to the phloem, thus lowering its concentration in the xylem stream and promoting nitrate transport to the younger leaves *via* the phloem (Hsu and Tsay, 2013).

The re-allocation of nitrate from source to sink tissues is of pivotal importance for improving the plant growth also under high nitrate concentration. NRT1.7, another NO_3_^–^ transporter, is involved in the loading of excess NO_3_^–^ present in the source leaves into the phloem, promoting NO_3_^–^ re-allocation to sink leaves. Under low NO_3_^–^, the *nrt1.7* mutant shows retardation of growth, demonstrating that NRT1.7-dependent NO_3_^–^ remobilization from source to sink tissues is essential to sustain plants’ growth (Chen K. E. et al., 2020).

Indeed, efficient uptake, assimilation, and re-mobilization of NO_3_^–^ are crucial for plant growth; however, at plant maturity, accumulation of NO_3_^–^ in the vacuole of some plants, especially leafy vegetables supplied with nitrate exceeding plant demand, may be considered dangerous (Martinoia et al., 1981). Vegetables represent the main source of the dietary NO_3_^–^ for humans, accounting for about 72–94% of the total intake (Dich et al., 1996). When NO_3_^–^ accumulation in the edible plant tissues exceeds the maximum residue levels (MRLs), it exerts serious ill-effects on human health (Gupta et al., 2017). In fact, it can be reduced to NO_2_^–^ by gastrointestinal microflora, leading to methemoglobinemia in children (Blue Baby Syndrome) (Aires et al., 2013; Colla et al., 2018; Kyriacou and Rouphael, 2018). Nitrite can react with amines and amides forming N-nitroso compounds (NOCs), categorized as “probably carcinogenic to humans” and linked to nasopharyngeal, esophageal, gastric, and colon cancers (Santamaria, 2006; Colla et al., 2018). Therefore, NO_3_^–^ content must be accurately monitored in leafy vegetables and composed lower than the limits imposed by EU regulation no. 1258/2011 (Giro and Ferrante, 2016).

## Chloride Interactions With Nitrate Uptake

Cl^–^ in excess can strongly reduce NUE specifically interfering with its uptake, transport, and loading into the root xylem, since it uses the same anion channels used by NO_3_^–^ (Diatloff et al., 2004; Carillo et al., 2005). The species’ sensitivity to salinity can be related to the Cl^–^-specific capacity of interference with their NO_3_^–^ uptake systems (Leidi and Lips, 2004). The Cl^–^-dependent reduction of cellular concentrations of NO_3_^–^ may indirectly downregulate the internal demand of NO_3_^–^ and consequently its uptake (Glass et al., 2002; O’Brien et al., 2016). In fact, as mentioned above, NO_3_^–^ may induce the expression and transcription of genes involved in its assimilation and transport, in addition to the genes involved in the energy and carbon metabolism (Galangau et al., 1988; Foyer et al., 1998; Goel et al., 2016; Zhao et al., 2018). Moreover, the decrease of NO_3_^–^ levels may cause the proteolysis of plastid proteins and the remobilization of metabolites (including amino acids) from old to young leaves, quickening the yellowing and senescence of older leaves (Soltabayeva et al., 2018; Carillo et al., 2019a).

When Cl^–^ decreases the NO_3_^–^ transport to the root xylem, its loading to shoot is increased simultaneously, determining the presence of toxic Cl^–^ levels that further impair the plant metabolism (Munns and Tester, 2008; Carillo et al., 2019a). Mild to moderate concentrations of Cl^–^ may be toxic, exerting more severe ion imbalance and hyperosmotic stress than that of Na^+^ in several horticultural species, with consequent reduction of plant growth and yield (Colla et al., 2013; Cirillo et al., 2019). In fact, at a concentration of 4–7 mg g^–1^ DW, Cl^–^ may be more toxic than sodium for Cl^–^-sensitive species, like herbaceous perennial plants (Cirillo et al., 2019), and at concentrations of 15–50 mg g^–1^ DW, it also proved to be toxic for Cl^–^-tolerant species if abruptly applied to the soil in a short time (Tavakkoli et al., 2010; Colmenero-Flores et al., 2019). Indeed, Cl^–^, as an essential micronutrient, at concentrations lower than 4 mg g^–1^, is involved in turgor and pH regulation and may act as a counteranion in the stabilization of membrane potential, a regulator of cytosolic enzymatic activities, and a co-factor of the photosynthetic water-splitting complex (White and Broadley, 2001; Geilfus, 2018). For this reason, under low Cl^–^ levels, this ion is actively uptaken by a secondary active Cl^–^/2H^+^ symport (Felle, 1994). However, recent reports have shown that prolonged exposures to nutrient solutions containing Cl^–^ at concentrations of 4–5 mM may cause a gradual non-toxic accumulation of Cl^–^ at values ranging between 25 and 50 mg g^–1^ DW (macronutrient levels), which still allows plants to grow without apparent stress symptoms (Colmenero-Flores et al., 2019). Raven (2016) and Franco-Navarro et al. (2016) had already reported that the application of Cl^–^ at 1–5 mM concentrations could help plants to maintain positive turgor pressure, regulate osmotic potential, and decrease stomatal conductance and transpiration, while improving water use and photosynthetic efficiency. Wege et al. (2017), reviewing the different routes taken by Cl^–^ in plants, suggested that the energy costs associated with uptake and storage of Cl^–^ in the vacuole for turgor maintenance are lower than those associated with NO_3_^–^ because Cl^–^ does not require the expense of ATP for proton gradient. Franco-Navarro et al. (2019) showed that Cl^–^, as a beneficial macronutrient, stimulated the formation of larger leaf cells with a lower stomatal density, thus indirectly decreasing the stomatal conductance and water consumption. At the same time, the increase in the surface area of chloroplasts exposed to the intercellular airspace of mesophyll cells facilitated CO_2_ exchanges and photosynthetic performance (Franco-Navarro et al., 2019). This new finding of Cl^–^ as a beneficial macronutrient has therefore been confirmed by several studies and has been included in the fourth edition of the Marschner’s Mineral Nutrition of Higher Plants book (Rengel et al., 2022).

When Cl^–^ is in excess, it is passively transported into the root cortical cells and the xylem by anion channels such as the NO_3_^–^ transporter NPF7.3 (Lin et al., 2008) and the S-type anion heteromeric channel SLAH1/SLAH3 (Qiu et al., 2016). High Cl^–^ concentrations at the leaf level turn out less controlled and more dangerous than those of sodium due to the lower capacity of leaf blades to exclude Cl^–^ (Munns and Tester, 2008; Colla et al., 2013) and its limited basipetal phloem transport toward the roots (Munns, 2002; Geilfus, 2018). When Cl^–^ is accumulated in high concentration in the leaf tissues, it initially decreases the apoplast osmotic potential interfering with the cellular water relations (Geilfus, 2018). Thereafter, it diffuses into the symplast by using anion (e.g., nitrate and phosphate) uptake symporters competing with these beneficial nutrients for the uptake within the cell (Carillo et al., 2005; Griffiths and York, 2020). High levels of cytosolic Cl^–^ exceed the Cl^–^ homeostatic control, causing a higher efflux of this ion into the chloroplasts and mitochondria, thus impairing the photosynthetic and mitochondrial electron transport chains and causing ROS formation (Tavakkoli et al., 2010). In these conditions, older leaves, at first, start showing necrosis symptoms at the leaf margins and tips (Ayers and Westcot, 1985; Geilfus, 2018). If the Cl^–^ stress is prolonged, necrosis spreads toward the middle of the expanded leaves, which do not work anymore as a source of photosynthates with a consequent loss of younger leaves too (Goodrich et al., 2009).

Recently, it has been found that the addition of a small molecule like omeprazole (OMP), a selective proton pump inhibitor of human gastric parietal cells H^+^/K^+^-ATPase (Van Oosten et al., 2019), can alter NO_3_^–^/ Cl^–^ homeostasis in the plant tissues under salinity, allowing plants to overcome the negative effects of Cl^–^ stress. Rouphael et al. (2018) suggested that OMP in tomato plants could trigger signal transduction pathways mediated by endogenous phytohormone or calcium that can activate sub-traits conferring Cl^–^ salinity tolerance. ABA, even when not able to regulate Cl^–^ root uptake or its compartmentalization in vacuoles of root cortical cells (Li et al., 2017b), can interact with and/or be transported by a specific root NO_3_^–^ transporter, encoded by the *AtNPF2.5* gene, belonging to the Nitrate Excretion Transporter (NAXT) subfamily that can operate Cl^–^ excretion from the root cortical cells plasma membrane under salinity (Li et al., 2017a). OMP could be responsible for regulating the expression of the *AtNPF2.5* gene, thus modulating the root cell Cl^–^ extrusion in the presence of ABA. Carillo et al. (2019b) have also hypothesized that OMP could be involved in a specific epigenetic single missense modification of a member of the family of the CLC anion transporters, CLCa, usually involved in the compartmentalization of NO_3_^–^ in the vacuoles of the root cells (Wege et al., 2010). This mutation could change Cl^–^ over NO_3_^–^ selectivity of CLCa transporter, inducing Cl^–^ compartmentalization in the root vacuoles while decreasing the loading of this toxic ion to leaves (Wege et al., 2010). In salt-stressed basil plants treated with OMP, an increase of NO_3_^–^, potassium levels and leaf area/expansion, and fresh yield were observed (Carillo et al., 2019b). It is possible that the exclusion of Cl^–^ from the cytosol of the root cells and the consequent membrane depolarization may activate an outwardly rectifying non-selective cation channel (NORC), first identified in the xylem cells of barley roots (Wegner and Raschke, 1994), which enable the passive non-selective transport of NO_3_^–^ and K^+^ to xylem, accelerating the transport of these ions to shoots.

## Nitrate Accumulation and Chloride Eustress

As mentioned above, NO_3_^–^ accumulation in leafy vegetables at maturity should be avoided. Nitrate accumulation in leafy vegetables may depend on genetic material and plant physiological condition, cultivation practices, and amount, timing, and form of NO_3_^–^ application [European farmers traditionally rely on NH_4_NO_3_ and Ca(NO_3_)_2_], as well as environmental conditions (light intensity, temperature, drought and/or salinity influencing water-use efficiency, and CO_2_ uptake) (Cantliffe, 1973; Escobar-Gutiérrez et al., 2002; Rouphael et al., 2018). Indeed, adopting practices to finely control/limit NO_3_^–^ content in leafy vegetables without impairing the plant growth and yield could add value to the vegetable products and improve the use of N fertilizers while reducing or preventing pollution (Santamaria, 2006; Anjana and Iqbal, 2007). In particular, salinity eliciting has been considered an effective strategy to decrease NO_3_^–^ accumulation in the leafy vegetables, thanks to the antagonism between Cl^–^ and NO_3_^–^ discussed above (Rubinigg et al., 2003; Colla et al., 2018; Rouphael and Kyriacou, 2018; Rouphael et al., 2018). The reduction and partial replacement of NO_3_^–^ with Cl^–^ in the nutrient solution may be also facilitated by using soilless/hydroponic cultivation, which allows to fine-tune the concentration of nutrients in the cultivation media (Rouphael and Kyriacou, 2018). In fact, decreasing the NO_3_^–^: Cl^–^ ratio in growth media for several days or weeks before harvest may reduce NO_3_^–^ accumulation in the edible plant parts (Rubinigg et al., 2003; Borgognone et al., 2016; Tabaglio et al., 2020). In particular, it has been found that accurately modulating the NO_3_^–^: Cl^–^ ratio of the nutrient solution may allow in reducing the NO_3_^–^ content in the leafy vegetables without abruptly modifying the ionic strength of the culture or fertigation media and therefore without causing N limitation or starvation (Carillo et al., 2019a; Table 2). Clearly, decreasing the NO_3_^–^: Cl^–^ ratio may alter the morpho-physiological and qualitative features of salt-sensitive crops; however, a mild to moderate salinity stress (eustress) may decrease leaf NO_3_^–^ accumulation, while also inducing the synthesis and accumulation of bioactive compounds (Akula and Ravishankar, 2011; Lucini et al., 2016; Woodrow et al., 2017; Kyriacou and Rouphael, 2018), and can increase the plant antioxidant response and hardening (Kim et al., 2008; Carillo et al., 2020). However, it has been suggested by Rosales et al. (2020) that Cl^–^, instead of impairing NO_3_^–^ uptake and transport, facilitates its assimilation, improving NUE in tobacco. Probably, the efficient and inexpensive compartmentalization of Cl^–^ in the vacuole prevents the storage of nitrate and promotes its reductive assimilation (Wege et al., 2017). Accordingly, Neocleous et al. (2021) found that replacing one-third of the standard recommended NO_3_^–^ supply with Cl^–^ in closed hydroponic systems determined a 2-fold increase of tomato NUE while decreasing NO_3_^–^ losses to one-half without affecting the fruit biomass production. Therefore, regardless of whether Cl^–^ is considered a nitrate antagonist or a beneficial macronutrient for NUE, it is important to finely modulate its dose for decreasing the NO_3_^–^ accumulation in leaves or improving its uptake and assimilation without decreasing the growth and productivity of the plants, thus tuning up a critical equilibrium called *sectio divina* (Rouphael and Kyriacou, 2018; Giordano et al., 2019; Carillo et al., 2020). In fact, Giuffrida and Noto (2009) observed that NO_3_^–^ in lettuce leaves decreased linearly with the increase of NaCl salinity (from 2.8 to 4.8 mS cm^–1^) and plant density, with negative effects on fresh yield. Borgognone et al. (2016) were able to reduce NO_3_^–^ accumulation in cardoon leaves grown in floating raft culture by using a nutrient solution having a NO_3_^–^: Cl^–^ ratio of 20:80 in the last 5, 10, and 15 days before harvest without negatively impacting the yield. Rouphael et al. (2019) obtained a decrease in accumulation of NO_3_^–^ in leaves of green and red-pigmented perilla by applying a 10 mM NaCl eustress, and at the same time, this treatment enhanced polyphenols and therefore the antioxidant activity. Lettuce plants underwent a decrease in the leaf NO_3_^–^ content between 20 and 35 mM NaCl, which determined an increase in polyphenols but also a decrease in the growth and yield proportional to the increase in the salinity (Carillo et al., 2020, 2021; Conversa et al., 2021). However, Conversa et al. (2021) found that the endive plants showed a decrease in the antinutrient nitrate without a simultaneous effect on the yield even at 35 mM NaCl (3.5 dS m^–1^), probably due to the higher salt tolerance of this plant. Rosales et al. (2020) proposed that only when Cl^–^ is available at basal concentrations in soils, in the range of a micronutrient, nitrate is compartmentalized in tobacco leaf vacuoles to play an osmotic function instead of being assimilated.

**TABLE 2:** Application of Cl^–^ eustress for reducing NO_3_^–^ accumulation in leafy vegetables.

Species	Growth conditions	Treatments	Observed effects	References
Lettuce (*Lactuca sativa* L. var. Domino, Elvira, Daguan)	Nutrient Film Technique	554 g l^–1^ CaCl_2_ or 1,132 g l^–1^ KCl	Elimination of N-NO_3_^–^ and addition of Cl^–^ in the nutrient solution determines the release of NO_3_^–^ from vacuoles and its assimilation into amino acids	Urrestarazu et al., 1998
Green lettuce (*Lactuca sativa* L. var. longifolia Xanadu)	Floating system	2.8, 3.8, and 4.8 mS cm^–1^	Decrease of NO_3_^–^ but also of yield linear with an increase of salinity and plant density	Giuffrida and Noto, 2009
Green lettuce (*Lactuca sativa* L. cv. Paris Island) Red lettuce (*Lactuca sativa* L. cv. Sanguine)	Floating system	0, 5, 10, or 20 mM NaCl	Limited effect of salinity on NO_3_^–^ decrease probably due to different climatic conditions	Neocleous et al., 2014
Cardoon (*Cynara cardunculus* L.)	Floating raft system	NO_3_^–^:Cl^–^ ratio (80:20, 60:40, 40:60, or 20:80)	Decrease of NO_3_^–^ and total N and increase of antioxidant metabolites (e.g., phenols, flavonoids) in the leaves linear with Cl^–^ increase in the nutrient solution. No detrimental effects on growth even at the NO_3_^–^:Cl^–^ ratio of 20:80.	Borgognone et al., 2016
Green perilla (*Perilla frutescens* var. frutescens) Red perilla (*Perilla frutescens* var. crispa)	Peat/perlite (2:1)	Non-salt control, 10, 20, or 30 mM NaCl	Decrease of nitrate (but also of growth and yield) and increase of polyphenols in both green and red-pigmented perilla under 10 mM NaCl.	Rouphael et al., 2019
Green and red lettuce (*Lactuca* sativa L. var. acephala)	Floating raft system	1, 10, 20, and 30 mM NaCl	Decrease of NO_3_^–^ only under 30 mM NaCl, a salinity concentration highly affecting plant fresh yield. NO_3_^–^ decrease probably due to reduction in plant growth and development.	Carillo et al., 2020
Green and red lettuce (*Lactuca* sativa L. var. acephala)	Floating raft system	Isosmotic concentrations of 20 mM NaCl, 20 mM KCl, or 13.3 mM CaCl_2_	Reduction of NO_3_^–^ in plant tissue at the second cut under NaCl and even more under CaCl_2_ treatments. A moderate decrease of fresh yield and an increase of lipophilic antioxidant metabolites.	Carillo et al., 2021
Lettuce (*Lactuca sativa* L. var. longifolia) Endive (*Cichorium endivia* L. var. var. latifolium Hegi)	Soilless cultivation system (floating or ebb and flow)	2.5, 3.5 dS m^–1^	NO_3_^–^ and slight plant dry biomass decrease in lettuce grown under the floating system linear with salinity increase. Slower NO_3_^–^ decrease in endive even under 3.5 dS m^–1^ probably for the higher salinity tolerance of this species.	Conversa et al., 2021
Lettuce (*Lactuca sativa* L. var. longifolia) Chard (*Beta vulgaris* L. ssp. vulgaris convar. cicla var. flavescens Dc.) Spinach (*Spinacia oleracea* L. var. America)	Perlite/vermiculite (4:6)	Mixture of SO_4_^2–^ + PO_4_^3–^ (control) or 5 mM Cl^–^ (salinity). For both treatments: NO_3_^–^ 5 mM (below the levels applied in the field by farmers.	The increase of the Cl^–^/NO_3_^–^ ratio reduced by 25–70% of leaf NO_3_^–^ content without impairing or increasing plant biomass.	Rosales et al., 2020

Considering that the accumulation of NO_3_^–^ is mainly responsible for the N oxides and nitrosamines in flue-cured tobacco during smoking, Cl^–^ eustress may also help reduce nitrosamine levels in cigarettes, thus improving the quality of these crops and contributing to prevent a large proportion of deaths due to lung cancer (Mirvish, 2007; Rosales et al., 2020).

## Conclusion and Future Perspectives

Enhancing the crop productivity and quality of the product together with taking care of environmental quality are urgent needs for the intermediate future. Meeting these two important goals presents a major sustainability challenge to growers, extension specialists, and researchers, which may be fostered by identifying the right source, rate, and time of N application. Such global NUE necessitates having a global view of the molecular and physiological basis of nitrate uptake, assimilation, and use in plants in the function of agricultural practices. Therefore, future attempts to modify and improve the plant productivity and/or quality through manipulation of the NUE will depend crucially on the knowledge that we gain from the new strategies of fertilization and management practices, that is timing, rate, and form of N application in relation with other nutrients and/or biostimulants. In addition, the combination of seed priming using novel, nitric oxide- and hydrogen sulfide-releasing (NOSH) hybrid molecules and foliar biostimulation using micro/macroalgae-derived extract (MAB), and vegetal-based protein hydrolysate can provide the required specific rapid induction responses since the early stage of cultivation and the wide-range long-term effects to improve NUE, profitability, and nutritional value of the vegetable crops. With regard to the nitrate accumulation and chloride eustress, the application of salinity *eustress* facilitated by hydroponics can reduce the accumulation of the anti-nutrient nitrate in the leafy vegetables. Finally, the comprehension of (i) genotype × management practices to enhance NUE and developing eco-friendly methods of cultivation with lower environmental impact and (ii) the molecular and physiological modes of actions responsible for the enhancement of NUE in vegetable crops under both open field and controlled conditions have to be encouraged.

## Data Availability Statement

The original contributions presented in this study are included in the article, further inquiries can be directed to the corresponding author.

## Author Contributions

Both authors listed have made a substantial, direct and intellectual contribution to the work, and approved it for publication.

## Conflict of Interest

The authors declare that the research was conducted in the absence of any commercial or financial relationships that could be construed as a potential conflict of interest.

## Publisher’s Note

All claims expressed in this article are solely those of the authors and do not necessarily represent those of their affiliated organizations, or those of the publisher, the editors and the reviewers. Any product that may be evaluated in this article, or claim that may be made by its manufacturer, is not guaranteed or endorsed by the publisher.
